# Control of gene expression in engineered mammalian cells with a programmable shear‐stress inducer

**DOI:** 10.1002/bit.27939

**Published:** 2021-09-20

**Authors:** Tobias Strittmatter, Paul Argast, Peter Buchman, Krzysztof Krawczyk, Martin Fussenegger

**Affiliations:** ^1^ Department of Biosystems Science and Engineering ETH Zurich Basel Switzerland; ^2^ Faculty of Science University of Basel Basel Switzerland; ^3^ Present address: Krzysztof Krawczyk, Novartis Pharma AG Basel CH‐4002 Switzerland

**Keywords:** mechanoperception, MscL, NFAT, Piezo1

## Abstract

In humans, cellular mechanoperception serves as the basis of touch sensation and proprioception, contributes to the proper programming of cell fate during embryonic development, and plays a pivotal role in the development of mechanosensitive tissues. Molecular mechanoreceptors can respond to their environment by mediating transient adjustments of ion homeostasis, which subsequently trigger calcium‐dependent alteration of gene expression via specific signaling pathways such as the nuclear factor of the activated T‐cells pathway. Although, mechanoreceptors are potential drug targets for various diseases, current techniques to study mechanically gated processes are often based on custom‐tailored microfluidic systems, which require special setups or have limited throughput. Here, we present a platform to characterize shear‐stress‐triggered, calcium‐mediated gene expression, which employs a programmable, 96‐well‐format, shear‐stress induction device to examine the effects of imposing various mechanical loads on mammalian adherent cell lines. The presented method is suitable for high‐throughput experiments and provides a large tunable parameter space to optimize conditions for different cell types. Our findings indicate that the device is an effective tool to explore conditions in terms of frequency, intensity, intervals as well as extracellular matrix composition alongside the evaluation of different combinations of mechanosensitive proteins for mechanically activated gene expression. We believe our results can serve as a platform for further investigations into shear stress‐controlled gene expression in basic research and drug screening.

## Graphical abstract

Mechanical induction of NFAT‐dependent gene expression in adherent cell lines using a high‐throughput shear stress device. Strittmatter et al. could show that CHO‐K1 cells are sensitive to mechanical stress and that shear stress sensitivity is influenced by the the extracellular matrix. Shear stress sensitivity could also be increased by the expression of mechano‐sensitive ion channels such as Piezo1 and MscL.

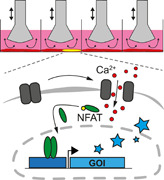

## 1 INTRODUCTION

Mechanical cues are omnipresent in the cellular environment and guide decisions of propagation and survival of prokaryotic and eukaryotic cells. In humans, these cues provide crucial information from the early stages of embryonic cell development to aid cell differentiation, and appropriate development of mechanosensitive tissues (Vining & Mooney 2017; Nonomura et al., 2018). Furthermore, they provide information on the environment (e.g., through senses of touch, hearing, or proprioception) (Geffeney & Goodman, 2012), and control various physiological parameters, for example, blood pressure (Xu et al., 2018).

Mechanosensitive proteins in the plasma membrane of cells are key mediators of mechanoperception (Geffeney & Goodman, 2012). Bacterial channels such as the mechanosensitive channel of small or large conductance (MscS or MscL, respectively) are simple exemplars. MscS and MscL each consist of two transmembrane domains and form heptameric (MscS) or pentameric complexes (MscL). They thereby established that mechanically gated pores are activated by local distortions of the membrane and serve as safety valves to protect bacteria from osmotic stress (Booth & Blount, 2012). These channels have been used as models to study the underlying principles of mechanoperception (Liu et al., 2009). In contrast, two of the major mechanosensors in humans, Piezo‐type mechanosensitive ion channel component 1 (Piezo1) and transmembrane channel‐like protein 1 (TMC1) comprise much larger and more complex structures. Piezo1 may play a key role in the sensation of touch, proprioception, and potentially blood pressure regulation (Saotome et al., 2018), while TMC1 may be a mediator of hearing (Pan et al., 2018). Mechanosensitive channels respond to mechanical cues by transient modulation of ion homeostasis that can trigger subsequent cellular responses through calcium‐dependent alteration of gene expression.

As a consequence of their involvement with various essential processes in human health and disease, mechanoreceptors are targets for the development of novel drugs (Xiao, 2020). However, current methods to tap into mechanically induced gene expression are mainly built around sophisticated microfluidic devices or focus on calcium transients rather than alterations of gene expression. Calcium transients alone, however, might not be a suitable means to investigate the full range of effects of the respective receptor.

To enable efficient screening of conditions for mechanically induced gene expression, we used a high‐throughput method to assess shear stress‐induced gene expression (Xu et al., 2018). We employed a 96‐well‐format shear stress induction device to explore ways of boosting the intrinsic as well as engineered mechanosensitivity of mammalian cells to trigger gene expression from a calcium‐responsive promoter. Parameters such as stimulus intensity, frequency, extracellular calcium concentration, and extracellular matrix composition, as well as the presence of accessory proteins, were assessed for their impact on gene expression. We believe this study provides tools and basic data for future explorations of mechano‐activated gene expression in mammalian cells.

## 2 MATERIALS AND METHODS

### 2.1 Cell culture

HEK293‐T cells and CHO‐K1 cells were cultured in 10 cm cell culture dishes (cat no.) in Dulbecco's modified Eagle's medium (DMEM, Gibco, #61965‐026) supplemented with 10 % fetal calf serum (FCS), 50 units/ml penicillin and 50 µg/ml streptomycin (Gibco, #15070‐063) in a humidified atmosphere containing 7.5 % CO_2_. The medium was supplemented with 150 µM L‐proline for culturing CHO‐K1 cells. All cell lines were split using trypsin‐EDTA (Gibco, #25300‐054) every 2 days or when they reached 70 % confluency, and reseeded at 1.5 million cells per dish. For cell culture experiments, cells were seeded at the indicated concentrations (15,000, 30,000, 45,000, or 60,000 cells per well) in 96‐well plates (Corning, #3599) or at 250,000 cells per well insix‐well plates (Corning, #3516) for generation of stable cell lines.

### 2.2 Plasmid generation

All plasmids were cloned by standard molecular cloning techniques. See Table 1 for a comprehensive list of plasmids and the genetic elements they carry. Details of the amounts of transfected plasmids for each experiment can be found in Table 2.

**Table 1:** Plasmid information

Plasmid name	Description	Reference
pmPiezo1‐IRES‐eGFP	Mammalian expression vector of mammalian Piezo1 coupled to internal ribosome entry site (IRES)‐mediated expression of enhanced green fluorescent protein (eGFP) from a constitutive promoter derived from human cytomegalovirus (P_CMV_) (P_CMV_‐Ca_V_1.2‐pA)	Coste et al. (2010)
pFS29	Mammalian expression vector for expression of red fluorescent protein mCherry from a constitutive promoter derived from simian virus 40 (P_SV40_) (P_SV40_‐mCherry‐pA)	Sedlmayer et al. (2018)
pDF101	Inert filler plasmid bearing a bacterial T7 promoter driving an inactive ribozyme (P_T7_‐SpAL‐sTRSVac)	Auslander et al. (2018)
pSEAP2‐Control	Constitutive SEAP expression vector (P_SV40_‐SEAP‐pA)	Clonetech
pTS391	Vector for stable integration of two expression cassettes flanked by insertion and recognition sites of Sleeping Beauty transposase (SB). Cassette one contains P_CMV_‐driven mammalian Piezo1 (mPiezo1). Cassette two comprises P_RPBSA_‐driven blue fluorescent protein (BFP) coupled to puromycin resistance marker (PuroR). (SB‐(1)CMV‐mPiezo1‐pA‐(2)RPBSA_BFP_p2a_PuroR‐pA‐SB)	This work
pTS395	Mammalian expression vector for SB100 Sleeping Beauty transposase driven by a constitutive promoter derived from human cytomegalovirus (P_CMV_) (P_hCMV_‐SB100‐pA)	This work
pTS775	Mammalian expression vector for expression of nuclear factor and activator of transcription c1 (NFATc1) from a constitutive promoter derived from human cytomegalovirus (P_CMV_) (P_CMV_‐NFATc1‐pA)	This work
pTS776	Mammalian expression vector for expression of nuclear factor and activator of transcription c2 (NFATc2) from a constitutive promoter derived from human cytomegalovirus (P_CMV_) (P_CMV_‐NFATc2‐pA)	This work
pTS1054	Mammalian expression vector for expression of bacterial mechanosensitive channel of large conductance (MscL) from a constitutive promoter derived from human cytomegalovirus (P_CMV_) (P_CMV_‐MscL‐pA)	This work
pTS2010	Reporter plasmid for NFAT‐induced expression of secreted nLuc reporter and blue fluorescent protein mTagBFP2 from a NFAT‐responsive synthetic promoter containing four response elements derived from the IL4 promoter (P_NFAT4_). (P_NFAT4_‐SS‐nLuc‐P2A‐mTagBFP2‐pA)	This work
pTS2258	Vector for stable integration of three expression cassettes flanked by insertion and recognition sites of Sleeping Beauty transposase (SB). The first cassette carries a reporter construct for NFAT‐induced gene expression of cytosolic nLuc reporter coupled to blue fluorescent protein (mTagBFP2). The second cassette contains infrared‐fluorescent reporter protein (iRFP) coupled to a resistance gene for puromycin (puroR) driven by a synthetic RPBSA promoter (P_RBSA_) and an ORF encoding nuclear factor and activator of transcription c1 (NFATc1) driven by an internal ribosome entry site (IRES). The third cassette enables expression of cytosolic firefly luciferase (fLuc) reporter for internal normalization of nLuc expression from a constitutive phosphoglycerate kinase promoter (P_PGK_). (SB‐(1)P_NFAT4_‐nLuc‐P2A‐mTagBFP2‐pA‐(2)RPBSA‐iRFP‐P2A‐PuroR‐IRES‐NFATc1‐pA‐(3)P_PGK_‐fLuc‐pA‐SB)	This work

**Table 2:** Transfection Details of the transfection procedure are given in the methods section

Figure	Plasmids used	ng per 96‐well	Goal of experiment
1c	pTS2010	50	Dependency of shear stress induced NFAT‐mediated gene expression on frequency
	pSEAP2‐Control	50	
	pDF101	Adjust to 200	
1d	mPiezo1‐IRES‐eGFP	10	Effect of mPiezo1 overexpression on shear stress induced gene expression
	pTS2010	50	
	pSEAP2‐Control	50	
	pDF101	Adjust to 200	
1e	pTS2010	50	Effect of timing of shear stress induced gene expression
	pSEAP2‐Control	50	
	pDF101	Adjust to 200	
2a	pTS2010	80	Test effects of NFATc1 or NFATc2 overexpression on NFAT responses under shear stress
	pVH288	40	
	pTS775	10/0	
	pTS776	1/0	
	pFS29	Adjust to 200	
2b	mPiezo1‐IRES‐eGFP/pTS1054/pFox8	10	Test coatings and overexpression of mechanoreceptors MscL and mPiezo1
	pFS29	Adjust to 200	

Transfection

Polyethyleneimine (PEI, 24765‐1, Polysciences Inc.) was used for the transfection of plasmid DNA in a ratio of PEI:DNA of 6:1. At 24 h prior to transfection, cells were seeded as described above. 200 ng or 1000 ng DNA was mixed with 150 µl or 500 µl of serum‐free DMEM and 1.2 µg or 6 µg PEI (1.2 µl or 6 µl of a 1 mg/ml stock solution) was added per well of a 96‐well plate or 6‐well plate, respectively. The mixture was incubated at room temperature for 5–10 min before adding it to the cells. Cells were incubated overnight (>12 h) under standard culture conditions. The medium was replaced by standard culture medium (as described above) the next morning and supplemented with CaCl_2_ or KCl as required by the experimental design.

Abbreviations: CMV, human cytomegalovirus; eGFP, enhanced green fluorescent protein; IRES, internal ribosome entry site; nLuc, NanoLuc luciferase; pA, polyadenylation site; P_CMV_, constitutive promoter derived from human cytomegalovirus; P_SV40_, constitutive promoter derived from simian virus 40; P_T7_, SV40, simian virus 40; PuroR, puromycine resistance gene; RPBSA, synthetic promoter comprising the RPL13a promoter fused to parts of the RPL41 gene; SS, secretion signal; SB100, sleeping beauty transposase SB100.

### 2.3 Generation of stable cell lines

For the generation of stable cell lines, cells were seeded in six‐well plates and transfected with donor vectors for the Sleeping Beauty transposase system as described above, using 600 ng of donor vector and 400 ng of pTS395 (P_hCMV_‐SB100X‐pA). Selection was done using 1 µg/ml of puromycin (Invivogen, #ant‐pr) for at least 10 days.

HEK‐293T cells stably expressing mammalian Piezo1 were generated by transfecting pTS391 and pTS395 while HEK‐293T and CHO‐K1 cells carrying the NFAT‐dependent nLuc reporter, NFATc1 expression cassette and constitutive expression of NLuc were generated using pTS2258 and pTS395.

### 2.4 Assessment of reporter gene expression

Activity of the reporter protein, secreted alkaline placental phosphatase (SEAP), was measured as previously described (Berger et al., 1988). In short, supernatant from cell culture experiments was heat‐treated for 30 min at 65°C to inactivate endogenous phosphatases. Approximately 20 µl of heat‐treated supernatant was mixed with 60 µl of water and 80 µl of two‐times assay buffer (1 M diethanolamine pH 9.8, 0.5 mM MgCl_2_, and 10 mM L‐homoarginine). The reaction was started by the addition of 20 µl substrate solution (120 mM p‐nitrophenyl phosphate in 2x assay buffer). Reporter activity was assessed by measuring the reaction kinetics at 37°C, by following substrate turnover at 405 nm with an Infinite M1000 microplate reader (Tecan Trading AG).

NanoLuc luciferase (NLuc) activity in the cell culture supernatant was recorded as recommended by the manufacturer. The 7.5 µl of Nano‐Glo® Assay Reagent was mixed with 7.5 µl of supernatant per well of a 384‐well plate and luminescence was measured with an Infinite M1000 microplate reader (Tecan Trading AG) using an integration time of 100 ms.

NanoLuc luficerase (NLuc) activity and firefly luciferase (FLuc) activity were measured in the same cell lysates using Nano‐Glo® Luciferase Assay (Promega) and ONE‐Glo™ Luciferase Assay (Promega) Kits. The culture supernatant was aspirated and replaced with 100 µl of one‐time Passive Lysis Buffer (Promega), followed by incubation for up to 30 min at room temperature with shaking at 400 rpm. About 7.5 µl of Nano‐Glo® or One‐Glo™ Assay Reagent was mixed with 7.5 µl of lysate per well of a 384‐well plate. Luminescence was recorded with a 100 ms integration time on an Infinite M1000 microplate reader (Tecan Trading AG).

### 2.5 Statistical analysis

No statistical analysis was performed. Data are presented as bar graphs representing means ± *SD* of *N* = 3 or *N* = 6 biologically independent samples.

## 3 RESULTS AND DISCUSSION

**Figure 1:**
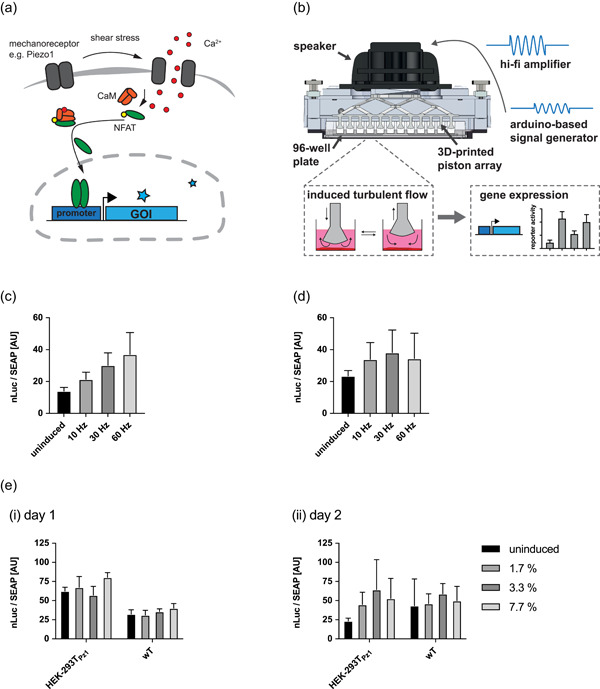
(a) Schematic representation of calcium signaling via the endogenous NFAT signaling pathway. Mechanosensitive channels such as Piezo1 in the plasma membrane trigger elevate cytosolic calcium, activating the trimeric calcineurin A (CnA)‐calcineurin B (CnB)‐calmodulin (CaM) complex. The activated complex binds to and dephosphorylates phosphorylated NFAT transcription factor through the phosphatase activity of CnA. Dephosphorylated NFAT can enter the nucleus and initiate transcription from NFAT‐responsive promoters. Additional channels such as calcium release‐activated channel (CRAC) or store‐operated channels (SOC) that would amplify the calcium signal are not depicted for the sake of clarity. (b) Shear stress‐inducing turbulent flow was generated by a 3D‐printed 96‐piston array attached to the membrane of a speaker controlled by a hi‐fi amplifier‐enhanced signal produced by an Arduino‐based signal generator. The up‐and‐down movement of pistons inside the wells induces turbulent flow, subjecting the cells to shear stress (left insert). Shear stress‐induced gene expression is assessed in terms of reporter gene expression (right insert). (c) Various frequencies and timings of shear stress were assessed for their effect on gene expression. To account for unspecific effects, a dual‐reporter setup was introduced using NFAT‐responsive expression of secreted NanoLuc luciferase (NLuc) and constitutive expression of SEAP from a weak constitutive promoter derived from the simian virus 40 (P_SV40_). By normalizing NLuc activity to SEAP expression, a more robust signal was generated. A clear dose‐dependent increase of NFAT activity was seen in HEK‐293T cells upon stimulation with the piston device. Induction was done at intervals of 5 s every 5 min at the indicated frequencies for 24 h and compared to corresponding uninduced samples. (d) Mechanosensitive channel Piezo1 was overexpressed in HEK‐293T cells grown for an additional 24 h after transfection under standard culture conditions to allow for proper expression of the channel before stimulation, as done in (c). (e) To ensure a more homogenous expression of Piezo1, we generated a HEK‐293T cell line stably expressing Piezo1 (pcTS1). Induction of the cell line either (i) directly after transfection or (ii) after 24 h of regeneration was assessed. All values are mean ± *SD*, *N* = 3 biologically independent samples. NFAT, nuclear factor of activated T‐cells; *SD*, standard deviation

To sense the mechanically induced increase in cytosolic calcium concentration, we employed a synthetic calcium‐inducible promoter based on NFAT‐responsive elements from the interleukin 4 (IL4) promoter cloned 5ʹ of a mammalian minimal promoter (Xie et al., 2016) driving expression of secreted NanoLuc luciferase (NLuc) (Figure 1a). Secreted human alkaline phosphatase (SEAP) was used as a constitutive reference to compensate for potential unspecific effects caused by the application of shear stress.

Generation of shear stress on whole‐cell populations was achieved using a custom‐made device inspired by a similar appliance used for calcium imaging to screen short hairpin RNAs targeting mechanosensitive proteins involved in calcium signaling (Xu et al., 2018). It consists of a 3D printed array of 96 pistons adapted for a regular 96‐well format, glued to the membrane of a loudspeaker (Figure 1b). The loudspeaker is controlled by a signal generator based on an Arduino controller coupled to an operational amplifier and a hi‐fi stereo amplifier. When the device is running, the pistons move up and down inside the cell culture wells to generate turbulent flow and hence shear stress on the bottom of the wells. Initial tests using the same device in combination with genetically encoded calcium indicator (GECI), GCaMP6s, in cultured mouse neurons provided the foundation for the present study by recording evoked transient calcium signals in response to mechanical stimulation (Gaub et al., 2020).

We used the piston device to apply shear stress with 10 Hz, 30 Hz, or 60 Hz to HEK‐293T cells and compared the NLuc response to untreated control. Indeed, we found increased reporter gene expression in response to mechanical load in a frequency‐dependent manner (Figure 1c). We next tested the transient expression of mechanoreceptor Piezo1 in HEK‐293T cells to boost mechanosensitivity and, hence, NFAT‐mediated gene expression. Transfecting cells with Piezo1 yielded higher overall reporter gene expression but lower fold switching compared with wild‐type cells (Figure 1d). In addition, Piezo1‐transfected cells showed higher sensitivity than wild‐type cells, reaching their maximal induction at 30 Hz compared to wild‐type cells which were not saturated at 60 Hz.

As we found the expression of Piezo1 to be slow and heterogeneous within a transiently transfected cell population, we next selected a HEK‐293T cell line stably expressing Piezo1 (HEK‐293T_Pz1_). We speculated that cell–cell adherence might be a major contributor to improved mechanosensitivity, and we, therefore, used this cell line both to confirm the ability of the prolonged preinduction cultivation used in previous experiments to boost induction fold and to test different induction protocols using induction times of 5 s every 5 min, 2.5 min or 1 min at 30 Hz (corresponding to duty cycles of 1.7%, 3.3%, and 7.7%, respectively). We found the inducibility of the Piezo1‐expressing cell line on Day 2 to be superior. Furthermore, higher duty cycles promoted higher fold switching in engineered as well as wild‐type cells (Figure 1e). As the expression of Piezo1 is thought to be constant over time, we speculate that the increased responsiveness on Day 2 points to the possible involvement of cellular adhesion complexes as well as extracellular matrix components that require more time to develop (Jiang et al., 2021).

**Figure 2:**
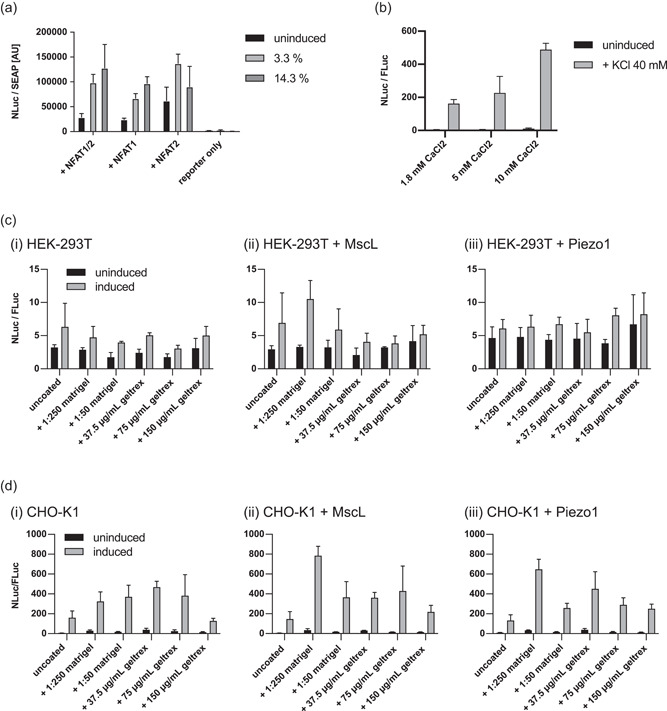
(a) NFATc1 and NFATc2 were tested for enhanced shear stress‐induced gene expression as single components or in combination (NFAT1/2) using 5 s stimulation each 2.5 min or 5 s stimulation each 0.5 min (3.3% or 14.3% duty cycle, respectively). To improve the robustness of the system and to streamline future experiments, stable cell lines were generated by designing a vector construct comprising NFAT‐responsive NLuc expression and constitutive expression of firefly luciferase (FLuc) alongside constitutive expression of NFAT1c. (b) Stable cell lines were used to compare the responses to KCl induction or shear stress‐mediated gene expression at different concentrations of extracellular calcium. Stable (c) HEK‐293T or d) CHO‐K1 cell lines were grown on Matrigel or Geltrex to simulate different extracellular matrix (ECM) compositions and densities as well as to test the effects of additional expression of mechanoreceptors (ii) MscL or (iii) Piezo1. All values are mean ± *SD*, *N* = 3 biologically independent samples. NFAT, nuclear factor of activated T‐cells; *SD*, standard deviation

We next assessed how the expression of key mediators of the NFAT response, NFATc1, and NFATc2, affects shear stress‐induced activation of the NFAT pathway. We found that especially overexpression of NFATc1 improves fold switches of HEK‐293T cells in response to shear stress induction in a dose‐dependent manner in HEK‐293T cells (Figure 2a).

To simplify the experimental design, we created a reporter construct that contains the already established NFAT‐reporter cassette driving NLuc expression as well as a constitutively expressed firefly luciferase driven by a phosphoglycerate kinase promoter (P_PGK_), replacing the SEAP reporter used in previous assays. We also added to the construct a P_RPBSA_‐driven selection cassette that expresses a polycistronic RNA comprising an infrared‐fluorescent protein (iRFP) coupled to a puromycin resistance gene (puroR) via a p2a sequence but also contains the CDS for NFATc1, which is driven by an internal ribosome entry site (IRES) (1:P_NFAT4_‐nLuc‐P2A‐mTagBFP2‐pA‐2:P_RPBSA_‐iRFP‐P2A‐PuroR‐IRES‐NFATc1‐pA‐3:P_PGK_‐fLuc‐pA). By using cytosolic versions of the luciferase reporters, we aimed to reduce dependency on the secretory pathway to avoid artifacts caused by the expression of multiple membrane proteins.

We used a so generated polyclonal stable HEK‐293T cell line to assess the influence of extracellular calcium on the activity of the NFAT pathway. As standard Dulbecco's modified Eagle's medium culture medium contains 1.8 mM calcium, we tested medium containing 1.8, 5, or 10 mM calcium. We found that increased calcium promotes higher fold switching in HEK‐293T cells induced with KCl compared with the standard formulation (Figure 2b). Thus, in subsequent experiments, we changed the standard medium composition to include a final concentration of 10 mM calcium during shear stress induction.

Further experiments were conducted to characterize the shear stress responses of this stable HEK‐293T reporter cell line when grown on different surface coatings (Matrigel or Geltrex) or transfected with mechanoreceptors MscL or Piezo1 (Figure 2c). Induction was performed at 30 Hz with a 10‐s‐on‐20‐sec‐off protocol (33% duty cycle). Although surface coatings were ineffective in changing the inducibility in untransfected stable cells (Figure 2c, i), low‐density Matrigel coatings resulted in improved fold‐switching in cells transfected with MscL (Figure 2c, ii). However, this could not be reproduced in cells transfected with Piezo1 (Figure 2c, iii), possibly due to a shortened incubation time of 24 h posttransfection.

Strikingly different results were obtained in a similar set of experiments using an identically generated stable CHO‐K1 cell line. In this case, surface coatings resulted in increased performance even in untransfected cells, with 37.5 µg/ml Geltrex showing the highest inducibility, though 150 µg/ml Geltrex showed the lowest inducibility (Figure 2d, i). Stable CHO‐K1 cells showed a strong increase in inducibility when transfected with mechanoreceptors MscL (Figure 2d, ii) or Piezo1 (Figure 2d, iii), with low‐density Matrigel or 37.5 µg/ml geltrex providing the biggest increase, whereas responses from cells grown in uncoated wells showed no change.

This study provides a platform for the characterization of shear stress‐triggered, calcium‐mediated gene expression in mammalian adherent cell lines. Transient calcium signals are routinely measured with genetically encoded calcium sensors (GECIs) or chemical calcium indicators but these approaches fall short of providing information on the ability of such calcium signals to activate gene expression. Here, we show that a custom‐made device in combination with a reporter construct of the endogenous NFAT pathway enables the detection of shear stress‐induced calcium responses.

By employing Piezo1 and MscL we could demonstrate that our method can be used to assess mechanoreceptor‐mediated activation of gene expression. Additionally, plasma membrane channels involved in calcium signaling such as the l‐type calcium channel Ca_V_1.2 have already been used to activate gene expression in combination with electrical stimulation or depolarization of cells to treat diabetes and chronic pain (Krawczyk et al., 2020; Wang et al., 2018; Xie et al., 2016). Other potential actuators such as ER‐channels of the ryanoidin receptor (RyR) family have yet to be explored in this regard but could be speculated to synergize with that of Ca_V_. However, because of the sophisticated architecture of Ca_V_ channels and similar ion channels that involve multiple large subunits (Zamponi et al., 2015), studying their responses by means of transient expression might be challenging. Alternative reporters might be used to assess the contributions of alternative signaling pathways in this setup, for example, cytoskeleton‐mediated pathways as well (Kirby & Lammerding 2018; Zhang et al., 2015).

Intriguingly extracellular matrix‐simulating coatings such as Matrigel and geltrex strongly boosted inducibility when used in combination with mechanoreceptor MscL in HEK‐293T, or with MscL and Piezo1 in CHO‐K1 cells. This cell‐type‐specific effect most likely reflects differences in the expression of adhesion molecules in response to shear stress (Shyy & Chien, 2002); thus, greater induction of adhesion molecules in CHO‐K1 cells compared with HEK‐293 cells results in stronger adherence, which in turn leads to better transmission of mechanical forces (Jiang et al., 2021). In accordance with this idea, we found that the density, and hence elasticity, of the plate coating, has a major impact on shear stress responses, especially in the case of CHO‐K1 cells. This dependency on ECM might also be indicative of a potential mechanism of activation for MscL and Piezo1 receptors that should be taken into account in future experiments. In this regard, the dependency of mechanosensitivity on ECM has already been reported for Piezo1 in HEK‐293T cells (Gaub & Muller, 2017).

We believe that the method described here for evaluating shear stress‐mediated gene expression has potential applications beyond basic research. For example, the method could be applied for drug screenings targeting mechanosensitive signaling pathways and receptors to provide a more holistic model that includes the underlying signaling cascades on top of calcium transients. Furthermore, mechanosensitive circuits could not only be used in combination with exogenous stimulation but could also be employed to sense physiological parameters such as flow rate inside the vasculature. For example, in next‐generation medicine, cells that contain mechanosensitive gene circuits might be employed to detect aberrant levels of blood pressure in the context of cardiovascular diseases, or as a part of stent implants to report on or even to counteract harmful occlusion events.

## Data Availability

All data and materials will be available upon request.
